# G9a regulates tumorigenicity and stemness through genome-wide DNA methylation reprogramming in non-small cell lung cancer

**DOI:** 10.1186/s13148-020-00879-5

**Published:** 2020-06-17

**Authors:** Rajendra P. Pangeni, Lu Yang, Keqiang Zhang, Jinhui Wang, Wendong Li, Chao Guo, Xinwei Yun, Ting Sun, Jami Wang, Dan J. Raz

**Affiliations:** 1 Division of Thoracic Surgery, City of Hope National Medical Center, Duarte, CA 91010 USA; 2 Department of System Biology, Beckman Research Institute, City of Hope National Medical Centre, Duarte, CA USA; 3 The Integrative Genomics Core Lab, Department of Molecular Medicine, City of Hope National Medical Center, Duarte, CA USA; 4 Frey Medical Laboratory, Maoling Rd, Jinan District, Fuzhou, Fujian China; 5 Key Laboratory of Cancer Prevention and Therapy, Tianjin Medical University Cancer Institute and Hospital, National Clinical Research Center for Cancer, Tianjin, China; 6 Western University of Health Sciences, Pomona, CA USA

**Keywords:** G9A, NSCLC, Stemness, TICs, Genome-wide methylation array, DNA RNA sequencing, Epigenetic regulation, Therapeutic approach

## Abstract

**Background:**

Eukaryotic histone methyltransferases 2 (EHMT2 or G9A) has been regarded as a potential target for non-small cell lung cancer (NSCLC) therapy. This study investigated the regulatory roles of G9A in tumorigenesis and stemness in NSCLC. We isolated and enriched tumor-initiating cells (TIC) from surgically resected NSCLC tissues by FACS and sphere formation assays. We then knocked down G9A using shRNA and carried out genome-wide 850K methylation array and RNA sequencing analyses. We carried out in vivo tumorigenecity asssay using mice xenografts and examined G9A interactions with its novel target using chromatin Immunoprecipitation (ChIP).

**Results:**

We identified 67 genes hypomethylated and 143 genes upregulated following G9A knockdown of which 43 genes were both hypomethylated and upregulated. We selected six genes (*CDYL2*, *DPP4*, *SP5*, *FOXP1*, *STAMBPL1*, and *ROBO1*) for validation. In addition, G9A expression was higher in TICs and targeting G9a by shRNA knockdown or by selective inhibitor UNC0642 significantly inhibited the expression of cancer stem cell markers and sphere forming capacity, in vitro proliferation, and in vivo growth. Further, transient overexpression of FOXP1, a protein may promote normal stem cell differentiation, in TICs resulted in downregulation of stem cell markers and sphere forming capacity and cell proliferation in vitro indicating that the genes we identified are directly regulated by G9A through aberrant DNA methylation and subsequent expression. Similarly, ChIP assay has shown that G9a interacts with its target genes through H3K9me2 and downregulation of H3K9me2 following G9a knockdown disrupts its interaction with its target genes.

**Conclusions:**

These data suggest that G9A is involved in lung cancer stemness through epigenetic mechanisms of maintaining DNA methylation of multiple lung cancer stem cell genes and their expression. Further, targeting G9A or its downstream genes could be a novel therapeutic approach in treating NSCLC patients.

## Background

Lung cancer is a leading cause of cancer death in men and women in the USA and worldwide [1]. Non-small cell lung cancer (NSCLC) accounts for 85% of lung cancer cases [2, 3]. There have been important advances in lung cancer therapy, including targeted therapies and immunotherapy; however, resistance to therapy inevitably develops and new therapies are desperately needed [4]. Although the 5-year survival for NSCLC has incrementally improved recently, it remains only 18% [5, 6]. Previous comprehensive studies on lung tumors have reported driver mutations, aberration in RNA transcript, and key pathways, of which a number of such alterations were targetable for therapies [4, 7, 8]. One of the dominant mechanisms responsible for cancer therapy resistance is epigenetic-mediated heterogeneity of cancer cell populations, which may be pre-existing or acquired with cancer therapy, and leads to expansion of cancer cell populations that are drug tolerant [9]. Although there has been promising clinical data on use of epigenetic therapies in patients with NSCLC, use of epigenetic therapies has been limited by their relative non-specificity which has led to toxicities [9]. The development of therapies targeting specific epigenetic changes holds promise as a new category of treatments for NSCLC alone or in combination with other treatments to overcome drug resistance [10].

Epigenetic plasticity is one of the hallmarks of cancer playing a profound role on tumor initiation and progression [11]. Epigenetic regulations based on DNA methylation, non-coding RNAs, chromatin remodeling, and post-translational modifications play a crucial role on lung cancer tumorigenesis [12]. DNA methylation and subsequent silencing of tumor suppressor genes through their CpG island promoter methylation has been a critical component in initiation and progression of lung tumor initiation and progression [13]. In addition to DNA hypermethylation of various genes, epigenetic dysregulation of DNA methyltransferases, chromatin remodeling enzymes, microRNAs, and histone-modifying complexes are reported in initiation and progression of lung cancer [14]. Furthermore, lung cancer exhibits heterogeneity in methylation in genome-wide level correlating to their inverse expression status that shows the methylation is an important component of tumor heterogeneity [15]. DNA methylation occurrences of individual histone modifications at global level is also associated with cancer as well as clinical outcome of patients [16]. Due the reversible nature of the epigenetics marks, tumors that show altered epigenome profiling could be targeted by the therapies that may restore the global epigenetic alterations [17].

Cancer stem cells (CSCs) comprised of a cell population in tumor contributing to intratumoral heterogeneity that is a major hurdle in treating cancer [18]. CSCs exhibit higher tumor-initiating capacity and are capable of recapitulate the heterogeneity of the parent tumors [19]. Moreover, a tumor consists of subsets of CSCs that has varying properties including therapy resistance, relapse, migratory, invasive and metastatic phenotypes, and epithelial-mesenchymal transition [20]. In addition, CSCs confers resistance to conventional chemotherapy and the conventional chemotherapeutic treatment further enriches CSCs and increased tumor burdens [21, 22]. Similarly, the residual population of tumor cells that resulted from chemotherapeutic treatment is enriched in CSCs capable of tumor relapse [23]. Epigenetic reprogramming plays a crucial role in carcinogenesis through CSCs and targeting these epigenetic aberrations could help in reducing tumor relapse and improve patients survival [24]. Therefore, targeting epigenetic modifiers in CSCs to modulate cancer microenvironment niche and signaling pathways as well as to correct epigenetic lesions is an important therapeutic strategy to treat cancers [11, 25].

G9a (also known as EHMT2) is a histone methyltransferases localized in euchromatin region that has a primary role in mediating mono- and dimethylation of H3K9, i.e., H3K9Me1 and H3K9Me2 [26]. Previous studies have reported that G9A is overexpressed in a number of cancers, and its overexpression is found to be associated with enhanced proliferation and metastasis [27, 28]. Previous study has shown that G9A exhibited its oncogenic function by dysregulating cellular iron hemostasis in breast cancer [29], epigenetically regulating metastatic genes in hypoxic condition in ovarian cancer [30], inducing angiogenic factors to promote angiogenesis in cervical cancer [31], modulating genes associated with DNA replication and RNA processing in hepatocellular carcinoma [32], sensitizing glioma cells for temozolomide (TMZ) when glioma cells were treated by G9a inhibitor [33], inhibiting cell proliferations in biliary tract cancer (BTC) cells [34], and urinary bladder cancer (UBC) cells [35] when treated with G9A inhibitor. It is evident that G9A cross talks with DNA methylation machinery to contribute to DNA methylation changes in cancers. In our previous study, we showed that G9A epigenetically regulates APC2 and HP1α through DNA methylation in NSCLC and its downregulation decreases cell proliferations and xenograft tumor growth in vivo [36].

In this study, we investigated the role of G9A in stemness and tumor initiation through genome-wide epigenetic reprogramming in using patient-derived tumor-initiating cells in NSCLC. We have identified novel genes regulated by G9A through DNA methylation alterations that could be important downstream targets of G9A and could be used for therapeutic interventions.

## Results

### G9A is highly expressed in CD133^+^ cells compared to CD133^−^ cells in patient-derived TICs in non-small cell lung cancer

**Fig. 1:**
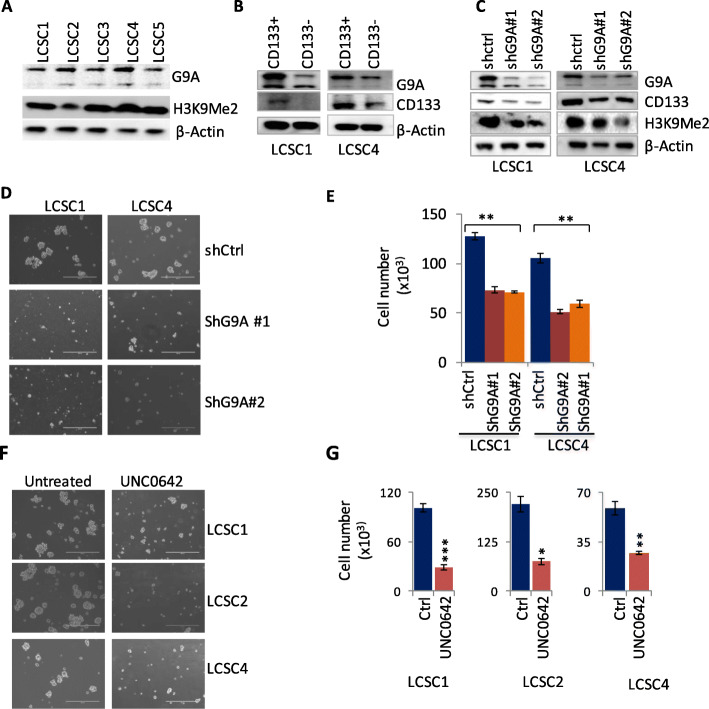
Targeting G9A decreased stemness and in vitro cell proliferation of tumor initiating cells (TICs) derived from NSCLC patients. **a** Basal level of G9A expression and corresponding H3K9Me2 level in tumor spheres of tumor-initiating cells (TICs) isolated from primary non-small lung cancer tissues determined by western blot. **b** G9A expression in FACS-sorted CD133^+^ and CD133^−^ cells in two TICs. **c** Expression level of CD133, and H3K9Me2 following G9A knockdown are shown. G9A knockdown resulted in decreased sphere forming (10X magnification) (**d**), and cell proliferation capacity (**e**) of TICs in vitro. Treatment of TICs with G9A inhibitor UNC0642 resulted in decreased sphere forming (× 10 magnification) (**f**) and cell proliferation (**g**) capacity in vitro. (For *t* test: *P < 0.05, **P < .01, and ***P < 0.001)

In order to investigate the role of G9A in TICs in lung cancer, we first isolated the cancer stem cell population and enriched a cancer cell subpopulation further from five primary lung adenocarcinoma tissues in serum-free medium for tumorsphere culture. The patient-derived tumorsphere isolated and grown in culture are shown in Supplementary Figure 1A. We examined a basal level of G9A expression and H3K9Me2 in these five patient-derived tumorspheres (Fig. 1a). The TICs were further sorted for CD133, one of the most widely used stem cell markers in order to enrich TICs with stem cells, and their CD133 expression-level was determined by Western blot (Fig. 1b, Supplementary Figure 1C). We observed that the CD133^+^ cells have higher levels of G9A expression compared to CD133^−^ cells (Fig. 1b, Supplementary Figure 1C). In addition, CD133^+^ cells have increased sphere-forming capacity compared to CD133^−^ cells (Supplementary Figure 1B).

We carried out stable knockdown of G9A using shRNAi, and found that G9A knockdown resulted in decreased CD133 expression and H3K9Me2 compared to its control (Fig. 1c, Supplementary Figure 1D-E). Further, we carried out sphere forming and cell proliferation assay. As shown in Fig. 1d, the two TICs (LCSC1 and LCSC4) knocked down with G9A showed decreased sphere-forming capacity compared to their control. Consistent with this finding, cells knocked down with G9A also had decreased cell proliferation (Fig. 1e). Further, we treated TICs cells with a selective G9A inhibitor UNC0642 [37]. The cells treated with UNC0642 showed decreased sphere forming and proliferation capacity (Fig. 1f, g). Cell proliferation capacity of TICs was also determined by measuring optical density of cells after treating cells for 72 h (Supplementary Figure 1E).

### G9A contributes to genome-wide DNA methylome and transcriptome changes in patient-derived TICs in non-small cell lung cancer

**Fig. 2:**
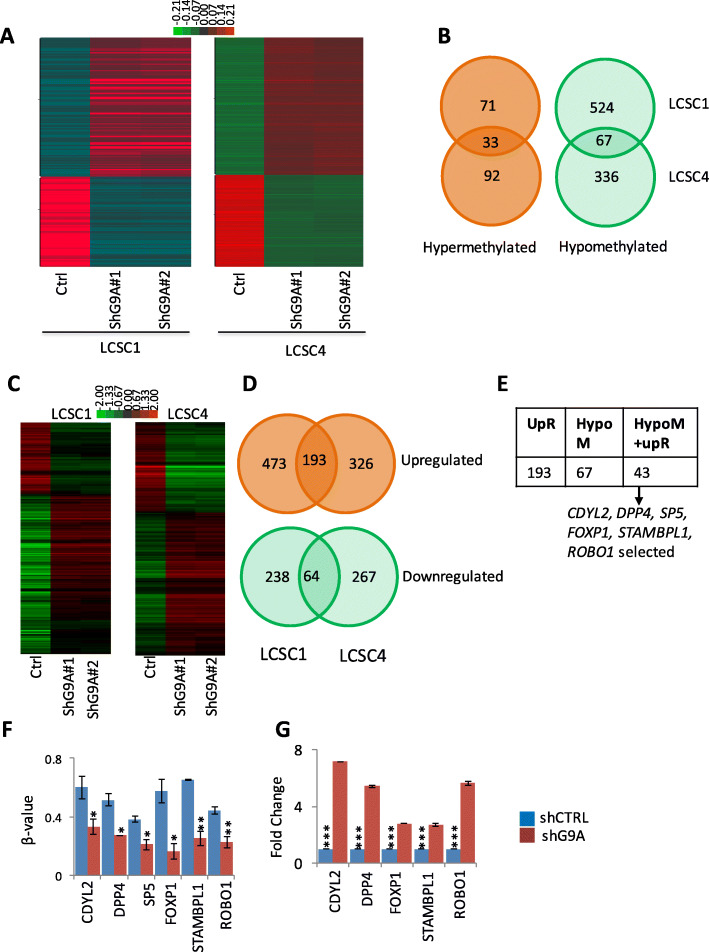
Impact of G9a knockdown on genome-wide methylome and transcriptome changes in patient derived TICs from NSCLC. Genome-wide methylation profiling (850K methylation array) of TICs, i.e., LCSC1 and LCSC4 to examine genes hypermethylated or hypomethylated following G9A knockdown as shown by clustering analysis (red-colored for hypermethylated, green-colored for hypomethylated) (**a**), G9A knockdown shows that a number of genes are commonly hypomethylated or are hypermethylated between LCSC1 and LCSC4, (**b**). **c** RNA sequencing from the TICs (LCSC1 and LCSC4) knocked down with G9A and their controls from LCSC1 and 4 shows that a number of genes are either upregulated (red-colored) or downregulated (green-colored) both in LCSC1 and LCSC4. **d** A number genes following G9A knockdown were also commonly downregulated or upregulated between LCSC1 and LCSC4. **e** Of 197 upregulated genes and 67 hypomethylated genes following G9A knockdown, 43 genes were common (whose methylation inversely correlates to their expression) and six genes *CDYL2*, *DPP4*, *SP5*, *STAMBPL1*, *FOXP1*, *ROBO1* were selected for downstream analyses. **f** Methylation (β value) obtained from 850K array and **g** mRNA expression (fold change) level of individual candidate genes obtained from RNA sequencing . (For *t* test: *P < 0.05, **P < .01, and ***P < 0.001)

G9A interacts with DNA methylation machinery to regulate DNA methylation in cancers [38]. We have previously shown that *APC2* promoter was methylated in control, whereas it was hypomethylated in lung cancer cell lines (H1299) that was treated with UNC0642 [36]. In order to investigate the roles of G9A in maintaining genome-wide DNA methylation level, we carried out genome-wide methylation analyses using HumanMethylation Epitect 850K array (850K-array) using genomic DNA from two patient-derived TICs (LCSC1 and LCSC4). The 850K array data analyses showed genome-wide methylation changes following G9A knockdown in LCSC1 and LCSC4 (Fig. 2a). First, we carried out unsupervised clustering of 850K data between control cells and knocked down cells in LCSC1 and LCSC4 (Supplementary Figure 2A, 2B). We further refined out genes based on methylation CpGs that had β value of > 0.35 as methylated and < 0.35 as unmethylated with β value difference of > 0.15 between them. We identified 104 genes in LCSC1 and 125 genes in LCSC4 that were hypermethylated following G9A knockdown compared to control, of which 33 genes were hypermethylated in both samples (Fig. 2b). Thus, 71 hypermethylated genes were unique to LCSC1, whereas 92 hypermethylated genes were unique to LCSC4. We identified 591 hypomethylated genes in LCSC1 and 403 hypomethylated genes in LCSC4 following G9A knockdown, and 67 genes were hypomethylated in both samples (Fig. 2b). Thus, 524 hypomethylated genes were unique to LCSC1, whereas 336 hypomethylated genes were unique to LCSC4.

In addition, our RNA sequencing data analyses showed genome-wide transcriptome changes following G9A knockdown in LCSC1 and LCSC4 (Fig. 2c). The data analyses resulted in 666 genes upregulated in LCSC1 and 519 genes upregulated in LCSC4, of which, 193 genes were common between them (Fig. 2d). Thus, our data resulted in 473 upregulated genes unique to LCSC1 and 326 upregulated genes unique to LCSC4. Similarly, of 302 and 331 genes downregulated in LCSC1 and LCSC4 respectively, of which 64 genes were commonly downregulated in both samples (Fig. 2d). Thus, 238 genes were downregulated uniquely in LCSC1 and 267 were downregulated uniquely in LCSC4. In order to identify novel genes that were methylated and silenced in TICs due G9A expression, we further carried out methylation-expression correlation from the set of 67 genes that were commonly hypomethylated and the set of 64 genes that were commonly upregulated in LCSC1 and LCSC4. This resulted in 43 genes that were hypomethylated and upregulated commonly in LCSC1 and LCSC4 (Fig. 2e). The methylation profiling of these genes are given in Supplementary Figure 2C. We choose our top 6 candidates based on their beta values differences (for methylation, Fig. 2f) and fold change of at least 2.5 between shCtrl and shG9A (Fig. 2g), biological function, and novelty. Therefore, our analyses identified novel candidate genes that are associated with stemness, and epigenetic regulation in lung cancer. Figure 2 f and g showed the methylation and expression profiling of selected six genes (*SP5*, *FOXP1*, *ROBO1*, *DPP4*, *CDYL2*, and *STAMBPL1*). These genes were hypomethylated and upregulated following G9A knockdown commonly between the two TICs (LCSC1 and LCSC4) compared to their controls.

### The genes regulated by G9A through methylation are involved in biological processes, pathways, and clinical prognosis of patients

**Fig. 3:**
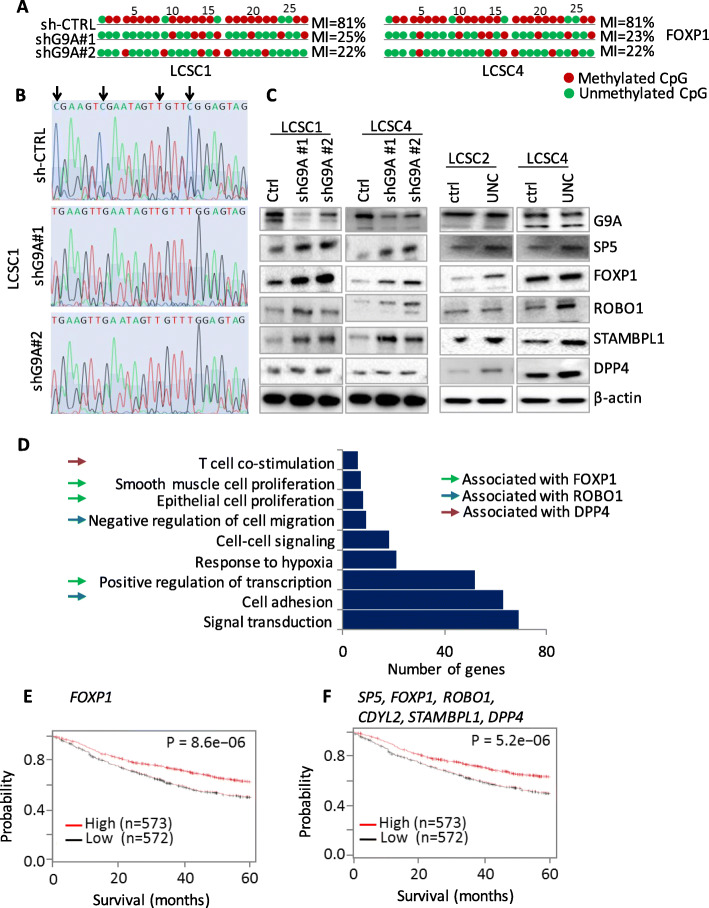
Validation of methylation status of candidates that were undergone DNA methylation and expression changes following G9A knockdown and their biological roles and clinical significance in NSCLC. **a** Methylation status of FOXP1 in TICs (LCSC1 and LCSC4) following G9A knockdown compared to their control as determined by Bisulphite PCR Sequencing (BS), chromatograms for the representative CpG dimers, and the methylation status (the red circles represent for methylated and the green circles represent for unmethylated CpG dimers) of the FOXP1 gene CpG dimers in TICs. The upper panel of chromatogram is for cancer cells transfected with control shRNA, the middle and lower panels are for TICs transfected with G9a shRNA. **b** Chromatogram of BS (as a representative example). **c** The protein encoded by genes methylated and silenced by G9A are upregulated following G9A knockdown (left panel) and treatment of TICs by G9A inhibitor UNC0642 resulted in increased expression of these proteins (*CDYL2*, *DPP4*, *SP5*, *STAMBPL1*, *FOXP1* and *ROBO1*). **d** Biological processes and pathways which are generated from the genes that are upregulated following G9A knockdown. Three genes *FOXP1*, *DPP4*, and *ROBO1* are associated with biological processes/pathways as shown. **e** Kaplan-Meier survival analysis of mRNA expression data of lung cancer indicates that high-expression of six candidate genes (*CDYL2*, *DPP4*, *SP5*, *STAMBPL1*, *FOXP1*, *ROBO1*) combined or **f** each individual gene (e.g., *FOXP1*) correlates to better clinical outcomes of patients in cancers (For both **e** and **f**, *n* = number of patients whose mRNA for respective genes were used for Kaplan-Meier analyses)

We carried out experimental validation of methylation status of our candidate genes using bisulphite sequencing (Fig. 3a, b and Supplementary Figure 3A and B). Bisulphite sequencing was carried out on promoter region or on a region around the differentially methylated CpG loci of the genes identified through 850K-array data analyses. The bisulphite converted samples (shCtrl vs. shG9A) amplified using semi-nested CoBRA (combined bisulphite and restriction analyses) PCR primers designed either on a promoter region or on a region around the differentially methylated CpG loci of candidate genes. The PCR-amplified DNA samples were purified and sequenced. The methylation level was determined by measuring methylation index (MI) between control samples and knocked down samples. MI is measured by calculating the ratio of methylated CpG (CG in the sequence is considered methylated and TG in the sequence is considered unmethylated) and the total number of CpG loci from the PCR amplified of respective genes. Our data shows that knockdown of G9A resulted in hypomethylation (decreased level of DNA methylation) of promoter of *FOXP1* (Fig. 3a, b), and *SP5* (Supplementary Figure 3B) in LCSC1 and LCSC4. Similarly, we carried out Western blot to examine expression of protein products of our candidate genes after G9A knockdown (Fig. 3c, left panel and Supplementary Figure 4) and after treatment with G9A inhibitor UNC0642 (Fig. 3c, right panel and Supplementary Figure 4). We identified that the hypomethylated genes (those with lower MI) were correlated inversely to their expression. Further, we examined wether the genes that are silenced in TICs by G9A through DNA methylation are also associated with biological processes and pathways. The databases for annotation, visualization, and integrated discovery (DAVID) [39] analyses on 992 genes (473 LCSC1, 326 in LCSC4 and 193 in common between them, refer to Fig. 3d) that were hypomethylated showed that the genes are associated with important biological processes and pathways. Among the six candidate genes, three genes (*FOXP1*, *ROBO1*, and *DPP4*) were associated with the biological processes and pathways identified through DAVID analyses (Fig. 3d).

In addition, our analyses on lung cancer databases using KM plotter tool [40] showed the higher expression of these genes correlate to better survival of lung cancer patients (Fig. 3e, Supplementary Figure 5). Further, our combined prognosis showed that the combined high expression of *SP5*, *FOXP1*, *ROBO1*, *DPP4*, *CDYL2*, and *STAMBPL1* is associated to patients’ better clinical prognosis of patients with lung cancer (Fig. 3f).

### G9K knockdown resulted in suppressed xenograft tumor growth in vivo and increase expression of its target genes

**Fig. 4:**
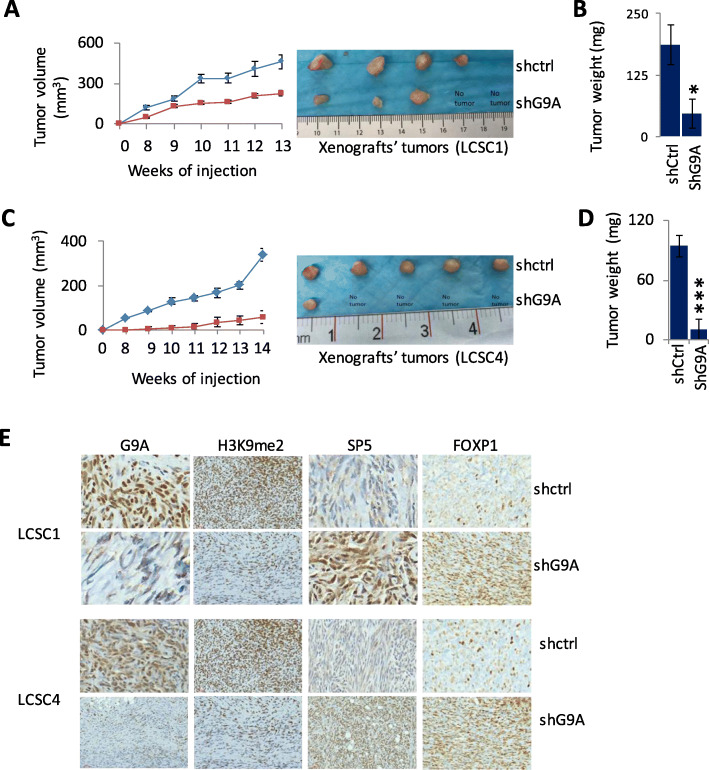
Knockdown of G9A in TICs (LCSC1 and LCSC4) strongly suppressed tumor growth in vivo. **a** Knockdown of G9A in LCSC1 significantly inhibited the tumor growth in mouse xenografts as shown in growth curves (left panel) and as resected tumors (four replicates for control and five replicates for knockdown, right panel). **b** Measurement of tumor weight (*P < 0.05 control vs. knocked down) shows the suppression of tumor growth is statistically significant. **c** Knockdown of G9A in LCSC4 significantly inhibited the tumor growth in mouse xenografts as shown in growth curves (left panel) and as resected tumors (right panel). **d** Measurement of tumor weight (***P < 0.001 control vs. knocked down) shows the suppression of tumor grown is statistically significant. **e** IHC staining of the tumors from the mouse xenografts shows reduced G9A expression (for validation of its knockdown) and H3K9Me2 level and increased expression of FOXP1 and SP5 both in LCSC1 and LCSC4 (× 20 magnification). (For *t* test: *P < 0.05, **P < 0.01, and ***P < 0.001)

We examined in vivo tumor growth of TICs following G9A knockdown. We implanted 100K TICs subcutaneously to examine xenograft tumors in mice. Compared to the control TICs (scrambled shRNAi), TICs knocked down with G9A significantly inhibited xenograft’s tumor growth. The tumor size was measured each week and pattern of tumor growth was recorded. The average tumor size in the mice injected with knocked down cells was significantly lower than the mice injected with control cells 6 weeks after the implantation both in LCSC1 (Fig. 4a) and for LCSC4 and (Fig. 4c). Moreover, the tumor weight in mice injected with knocked down cells was significantly lower than the tumor weight in the mice injected with control cells in LCSC1 (Fig. 4b) and LCSC4 (Fig. 4d). Further, immunohistochemistry (IHC) was used to determine the expression of target proteins from the xenograft tissues. IHC data showed that the mouse xenograft tissues have decreased level of H3K9Me2 protein and increased level of SP5 and FOXP1 both in LCSC1 and LCSC4 (Fig. 4e, Supplementary Table 4). Consistent with our in vitro data, G9A knocked down significantly restored the expression of G9A target genes from xenograft tissues which indicates that G9A contributes to DNA methylation and silencing of its target genes.

### G9K inhibitor UNC0642 resulted in suppressed xenograft tumor growth in vivo and increased expression of its target genes

**Fig. 5:**
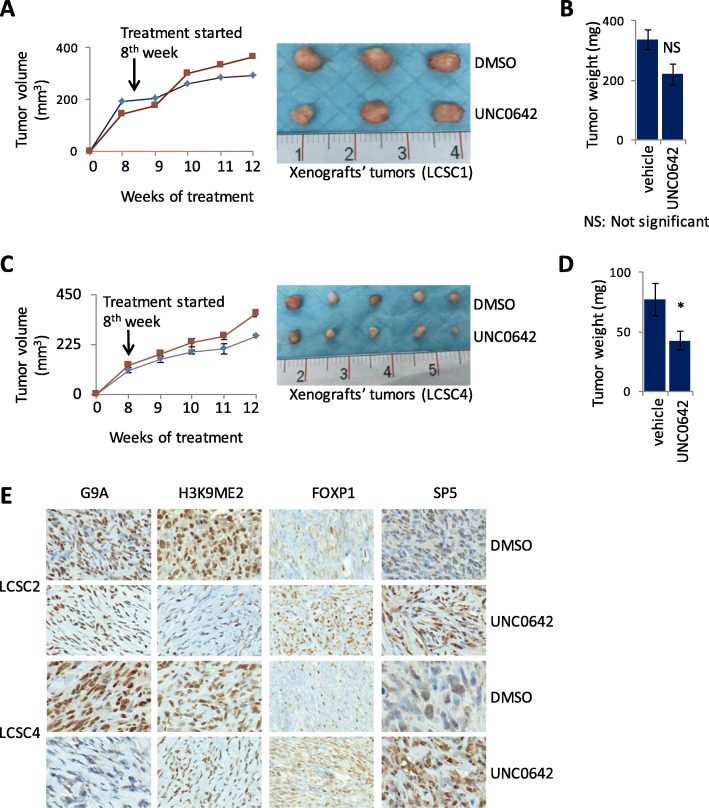
G9A inhibitor UNC0642 suppressed tumor growth of mouse xenografts (from LCSC2 and LCSC4). **a** UNC0642 treatment 10 mg/ml) inhibited LCSC2 xenografts growth as shown in growth curve (left panel) and as resected tumors (right panel). **b** Tumor weight in mice treated with control (vehicle) and 10 mg/ml UNC0642 shows a level of suppression of tumor growth in mice (*P* = NS; not significant). **c** UNC0642 treatment (10 mg/ml) inhibited LCSC2 xenografts growth as shown in growth curve (left panel) and as resected (right panel). **d** Tumor weight in mice treated with control (vehicle) and UNC0642 (10 mg/ml) shows tumor growth in treated mice is statistically significant (*P = 0.05). **e** IHC staining of the tumors from the mouse xenografts shows reduced G9A expression (for validation of its knockdown) and H3K9Me2 level and increased expression of FOXP1 and SP5 both in LCSC2 and LCSC4 (× 20 magnification). (For *t* test: *P < 0.05, **P < .01, and ***P < 0.001)

We examined anti-tumor effect of the G9A inhibitor UNC0642 in mouse xenograft models from two TICs (LCSC2 and LCSC4) and investigated the therapeutic efficacy of G9A inhibitor UNC0642 [37]. The spheres from LCSC2 and LCSC4 were split into single cells suspensions and 100,000 cells each from these TICs were injected subcutaneously to establish xenograft tumors in mice. For treatment, intraperitoneal (IP) injection was carried out. Treatment with UNC0642 injected at the dose of 10 mg/kg suppressed xenograft tumor growth in LCSC2 (Fig. 5a, b) and significantly supressed the growth in LCSC4 (Fig. 5c, d). The injection was started after 2 weeks of cells implantation and the tumor size was monitored every week. The average tumor size and weight of tumors in the mice treated with UNC0642 was lower than the mice treated with vehicle control. These results suggest that the UNC0642 inhibits tumor growth in a mice xenograft model. In addition, the xenograft tissues were fixed and analyzed by IHC to investigate if UNC0642 treatment in vivo results in upregulation of CDYL2, DPP4, FOXP1, STAMBPL1, ROBO1, and SP5 that are downregulated by G9A. Consistent with our in vitro data and in vivo knockdown findings, treatment with UNC0642 strongly decreased the level of H3K9Me2 methylation and increased the expression of SP5 and FOXP1 (Fig. 5e).

### Combined treatment of lung TICs with UNC0642 and 5-aza-2′-DC resulted in synergistic effects on suppression of sphere forming and cell proliferation capacity of TICs in vitro

**Fig. 6:**
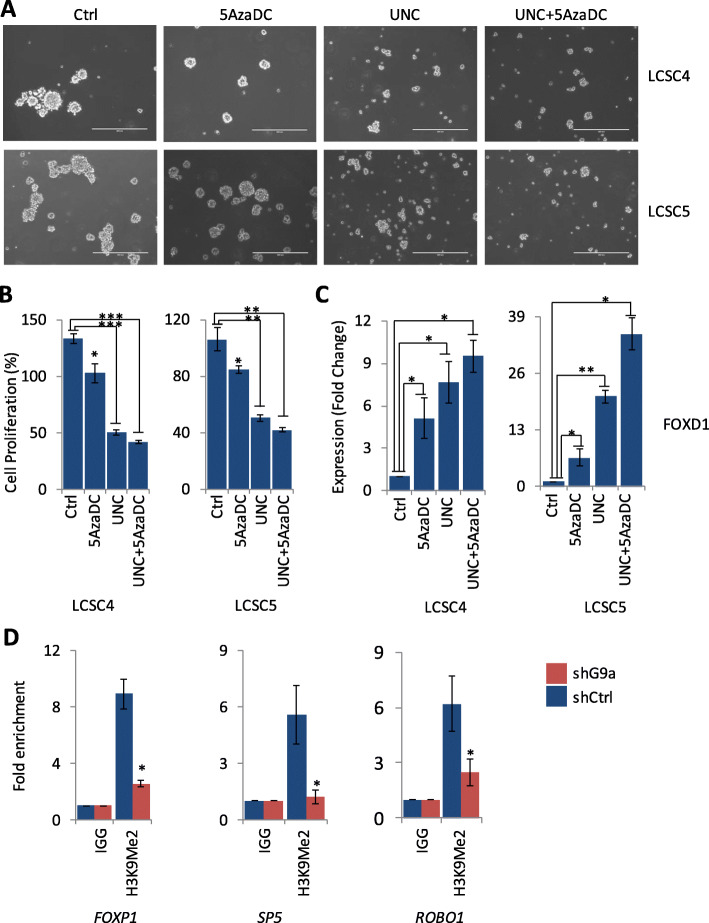
Effects of combined treatment of TICs with UNC0642 and 5-aza-2′-DC sphere forming and cell proliferation capacity of TICs and reactivating G9a target genes in vitro. **a** Sphere-forming assay on TICs (LCSC4 and LCSC5) following treatment with G9A inhibitor UNC0642 and DNA demethylating agent 5-aza-2′-DC alone and in combination compared to the control (× 10 magnification). **b** Cell proliferation assay and **c** qRT PCR of target genes to examine their mRNA level after the cells were treated by UNC0642, 5-aza-2′-DC and with combination of UNC0642 and 5-aza-2′-DC. **d** QPCR following chromatin immunoprecipitation assays on G9a target genes *FOXP1*, *SP5*, and *ROBO1*. (For *t* test: *P < 0.05, **P < 0.01, and ***P < 0.001)

We treated LCSC4 and LCSC5 with a combination of UNC0642 with 5-aza-2′-DC in order to investigate the synergistic effects on sphere forming and cell proliferation capacity of TICs as well as expression of G9a target genes. Compared to control cells, treatment of cells with 5-aza-2′-DC or UNC0642 alone resulted in decreased sphere-forming and cell-proliferating capacity of TICs (Fig. 6a, b). In addition, with a combination of 5-aza-2′-DC and UNC0642 resulted in more decreased sphere-forming and cell-proliferating capacity of TICs compared to control or treatments by 5-aza-2′-DC or UNC0642 alone (Fig. 6a, b). Similarly, treatment of TICs by 5-aza-2′-DC, UNC0642, or treatment with combination of 5-aza-2′-DC and UNC0642 resulted in G9a target genes expression at mRNA level as determined by qRT PCR (Fig. 6c, Supplementary Figure 6A-C). Our data shows that combined treatment with 5-aza-2′-DC and UNC0642 resulted in higher level of re-expression of G9a target genes compared to individual treatment by 5-aza-2′-DC or UNC0642.

### Chromatin immunoprecipitation assay shows that G9A interacts indirectly with the promoter of its putative target genes through H3K9me2

We performed chromatin immunoprecipitation (ChIP) using anti-H3K9me2 antibody followed by quantitative PCR using LCSC4 cells stably transfected with shCtrl and shG9a in order to examine interaction of G9a with its target genes. Our data shows that there is a specific dis-enrichment on promoter of *FOXP1*, *SP5*, *ROBO1*, *CDYL2*, and *DPP4* (Fig. 6d, Supplementary Figure 6D, E) from control (shCtrl) cells but not in shG9A cells. These data suggests that G9a interacts with its target genes through H3K9me2 and downregulation of H3K9me2 following G9a knockdown disrupts its interaction.

### Overexpression of G9A target gene FOXP1 resulted in decreased sphere-forming and proliferation capacity of TICs in vitro and downregulation of stem cells markers

**Fig. 7:**
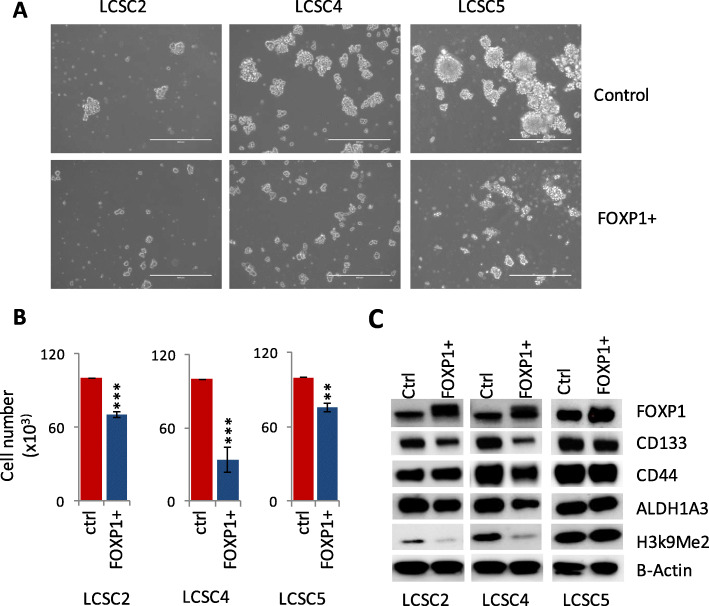
FOXP1 overexpression resulted in decrease in in vitro cell proliferation and sphere forming capacity of TICs as well as downregulation of H3K9Me2 and stem cells markers. **a** FOXP1 overexpression in TICs (LCSC2, LCSC4, and LCSC5) resulted in decreased sphere forming capacity (× 10 magnification), **b** decrease in cell proliferation capacity, and **c** downregulation of H3k9Me2 and stem cells markers CD44, CD133, and ALDH1A3. (For *t* test: *P < 0.05, **P < 0.01, and ***P < 0.001)

In order to investigate the role of the genes we identified on stemness and tumor growth, we intended to carry out functional validation of one of our G9A target genes in vitro. Carrying out functional validation of all of our target genes was beyond the scope of this manuscript; therefore, we chose FOXP1, a classical transcriptional factor and overexpressed it in three TICs (LCSC2, LCSC4, and LCSC5). After 48 h of overexpression of FOXP1, sphere-forming capacity (Fig. 7a) and in vitro growth (cell proliferation) of TICs (Fig. 7b) were decreased. Similarly, FOXP1 overexpression resulted in decrease in H3K9Me2 level and downregulation of three stem cell markers CD44, CD133, and ALDH1A3 in LCSC2, LCSC4, and LCSC5 (Fig. 7c, Supplementary Figure 7). These data shows that *FOXP1* functions as a tumor suppressor gene in NSCLC and is downregulated by G9A through DNA hypermethylation.

## Discussion

In this study, we investigated the role of G9A in stemness and tumorigenicity in NSCLC by examining genome-wide methylome and transcriptome changes following G9A knockdown in patient-derived TICs. Our data showed that G9A is upregulated in CD133^+^ cells compared to CD133^−^ cells from patient-derived TICs, and knocking down G9A downregulated CD133 in these cells. In addition, G9A knockdown resulted in decrease in both the proliferation and sphere-forming capacity of TICs. Similarly, treating the cells with the G9A inhibitor UNC0642 decreased CD133 expression as well as proliferation and sphere-forming capacity of TICs. These data are also consistent with our previous finding that G9A depletion downregulated the proliferation capacity of lung cancer cells [36].

Furthermore, we employed a novel approach to carry out genome-wide methylation and expression analyses in NSCLC TICs before and after G9A knockdown to determine the effects of G9a depletion on methylation-regulated gene expression. This study has identified a number of novel genes which are either upregulated or downregulated following G9A knockdown. In this study, we focused on those genes that were hypermethylated and upregulated when G9A was knockdown in TICs. Our genome-wide screening including methylation and expression correlation identified six genes; *CDYL2*, *DPP4*, *SP5*, *FOXP1*, *STAMBPL1*, and *ROBO1* that were both upregulated and hypomethylation following G9A knockdown, which we then validated for downstream analyses. Due to the DNA hypomethylation and subsequent overexpression of these genes following G9A knockdown, we hypothesized that these genes have tumor suppressor function in NSCLC that is regulated by G9a. In addition to experimental validation of methylation and expression level of these genes in TICs knocked down with G9A and their controls, we treated the TICs with G9A inhibitor UNC0642 and examined the expression of G9A target we identified. Consistent with our DNA methylation and RNA sequencing data, these genes were upregulated after treating cells for 72 h. The hypomethylation and subsequent upregulation of these genes following G9A depletion support the notion that G9A is an important regulator of DNA methylation of TICs in NSCLC through cross-talk with DNA methylation machinery.

Previous studies have reported that G9A coordinates DNA methylation by direct interaction with DNA methyltransferaase-1 (DNMT1) through H3K9me2 where G9a mediates H3K9me2 that helps to co-localize DNMT1and G9a during replication and cell division [38]. Genome-wide methylation analyses have been widely used to identify epigenetically dysregulated multiple genes in lung cancers [41, 42]. Similarly, the role of G9a in mediating H3K9me2 has been widely reported in previous studies [27, 36]. In this study, we have utilized a novel approach to elucidate DNA methylation reprogramming in TICs through histone methylation machinery or G9a. Reactivating epigenetically dysregulated genes through DNA methylation has been challenging due to the nonspecific nature of DNA methylation inhibitor such as 5-aza-2′-DC. This study has identified novel and common targets for both DNMT1 and G9a. Re-expression of G9a target genes through combined treatment of G9a inhibitor and 5-aza-2′-DC may indicate that a histone methylation and DNA methylation crosstalk could possibly be utilized for therapeutic purposes. Moreover, interaction of G9a with its target genes through H3K9me2 as shown through our chromatin immunoprecipitation data may indicate that these novel genes we identified could be used as therapeutic targets alone or in combination with G9ai and DNMTi. However, further mechanistic, functional, and therapeutic studies are necessary to investigate if these targets genes could be used as effective therapy for NSCLC. The genes we identified and validated are known cancer-related genes. Roundabout guidance receptor 1 (ROBO1), a transmembrane protein encodes protein that functions as a receptor for SLIT1, which together as a complex form an important regulator of Slit-Robo signaling pathways. Slit-Robo pathway has been associated with cancer development and metastasis [43, 44]. Previous studies have shown that ROBO1 is epigenetically silenced by promoter hypermethylation in breast, lung, and renal cancers [45]. Also, ROBO1 deletion is reported as one of the molecular alterations in small cell lung cancer, and SLIT-ROBO pathway genes are frequently altered and epigenetically regulated in breast, pancreas, and cervical cancers [46–49].

SP5, a transcription factor, is expressed predominantly in gastrodermal epithelial stem cells and maintains self-renewal of embryonic and neuromesodermal stem cells [50–52]. It is a target for WNT/β-catenin signaling and its regulation is maintained by WNT/β-catenin feedback loop [50]. In colorectal carcinoma cell lines (HCT1116), SP5 inhibits cell growth by upregulating P27, another tumor suppressor gene [53]. Previous studies reported genome-wide binding of SP5 on selected locations which is activated in normal stem-like cells, which however, contributes termination of transcriptional programs regulated by WNT signaling as feedback loop mechanism [54, 55]. Similarly, dipeptidyl peptidase 4 (DPP4), also known as CD26, is a cell surface glycoprotein having a role on T cell stimulation [56]. DPP4 regulates activities of various biomolecules such as cytokines and chemokines hence showing a dual roles as both tumor suppressor and activator in various cancers [57]. DPP4 overexpression is linked to prolonged survival of patients with mesothelioma, is hypermethylated and downregulated in cervical cancer, and seems to be regulated by C-myc in colon cancer [58–60].

Chromodomain Y like 2 (CDYL2) is a member of CDY-related mammalian gene family [61]. CDYL2 is reported to be a binding partner of H3K9Me3 in mouse embryonic stem cells (mESCs) associated with cancer and genomic stability [62]. Interestingly, CDYL2 has also been reported as a binding partner for methylated ARK(S/T) motif found in linker histone H1.4 and histone methyltransferases G9A [63].

Forkhead Box P1 (FOXP1) belongs to the forkhead box transcription factor family, and has known to have dual roles as a tumor suppressor gene and as an oncogene in multiple cancer types [64, 65]. FOXP1 deletions are found in squamous cell carcinomas of the lung, and expression of FOXP1 is associated with breast cancer development in poor prognosis in patients [4, 64, 66]. In addition, FOXP1 has been identified as a critical effector of PRMT5, an arginine methyltransferases regulating proliferation and self-renewal of breast cancer stem cells through a mechanism in which arginine PRMT5 recruitment to FOXP1 promoter facilitates H3K4me3 and H3R2me2 as well as SET1 recruitment [67]. Similarly, FOXP1 is associated with HIFs and androgen receptor (AR) in prostate cancer and downregulates AR-induced transcriptional activities and histone modifications in enhancer regions [68, 69]. In our study, overexpression of FOXP1 in TICs resulted in decreased proliferation and sphere-forming capacity of TICs in vitro, and a decrease in stem cells markers (CD44, CD133, and ALDH1A3) and H3K9Me2 compared to control cells, which is consistent with its tumor suppressor function in NSCLC in tumor initiation and progression.

We have shown that G9a is an important epigenetic regulator of oncogene expression in TICs derived from human patient samples. Moreover, we found that G9a is a key regulator of lung cell tumorigenesis and cell proliferation in TICs. These findings are important because TIC or cancer stem cells are thought to be largely responsible for metastasis and therapy resistance. Targeting G9a alone or in combination with other therapies may be a novel approach to treating patients with advanced lung cancer. Strengths of our study include our use of TICs derived from fresh surgically resected human lung cancer samples, utilization of genome-wide methylation and gene expression data, and validation of specific oncogenes regulated by G9a.

Further investigation is needed on the mechanism by which G9a interacts with DNMT1 to regulate DNA methylation. This may be important to identify additional therapeutic targets in lung cancer that disrupt this interaction. Although we used a specific G9a inhibitor UNC0642 in this study, current G9a inhibitors are not suitable for use in humans due to poor bioavailability and modest potency. Development of G9a inhibitors that are more potent and have favorable pharmacokinetics may lead to an effective therapy for lung cancer which might be used alone or in combination with standard therapies.

## Conclusions

In summary, we investigated the role of G9a in stemness and tumorigenicity and its role on genome-wide epigenetic reprogramming of NCSLC. Using patient-derived TICs, we showed that G9A is involved in NSCLC stemness by maintaining CD133 expression which further contributes to sphere forming and growth capacity of TICs and its inhibition resulted in decrease in stemness and tumorigenicity in vitro and in vivo. Further, we showed that knocking down G9A resulted in genome-wide methylation and expression of multiple genes which shows that it is a key epigenetic regulator of oncogene expression in TICs derived from lung cancer patient samples. One of the major challenges in treating lung cancer patients is thought to be metastasis to the other organs and therapy resistance that is largely attributed to CSCs or TICs. Our data with TICs derived from fresh surgically resected human lung cancer samples suggests that targeting G9A or its downstream genes could be a novel therapeutic approach in treating NSCLC patients.

## Materials and methods

### Patients and samples

#### TICs isolation and culture, sphere formation, and cell proliferation assays

We isolated putative tumor-initiating cells (TICs) from six cases of lung adenocarcinoma through surgical resection with curative intent without preoperative chemotherapy or radiation therapy at City of Hope. The details of clinical characteristics are also provided in Supplementary Table 1.

In order to isolate TICs, the tissues were placed in DMEM F12 basal medium immediately after the surgical resection. The tissue was minced and transferred into freshly prepared DMEM/F2 containing 400 U/ml collagenase IV (Gibco, USA). The cells were dissociated by incubating at 37 °C for 2 h with repeated shaking in every 10 min. The samples were mixed with ACK lysis buffer to lyse red blood cells with ACK lysis buffer (Gibco) in room temperature for 2 min. After a brief centrifugation and removing supernatant, the cell pellet was cultured in DMEM/F12 medium (Gibco) supplemented with 2% B-27 (Gibco, USA), 25 ng/ml FGF (Peprotech, USA), 25 ng/ml EGF (Peprotech, USA), 2 μg/ml heparin (EDQM, France), and 100 U/ml penicillin/streptomycin (Gibco, USA). The cells were then transferred to 10 cm ultra-low-attachment flask (company) for further expansion and experimentation. This study was reviewed and approved by the Institutional Review Board (IRB) 17196 of City of Hope National Medical Centre, and informed consent for the collection of tumor tissues for the study were obtained from all patients.

For in vitro sphere formation and cell proliferation assays, TIC-spheres were dissociated into single cells using StemPro accutase (ThermoFisher Scientific, USA), and 50,000–100,000 cells were seeded in 6-well plates. The cells were treated with 10 μM UNC0642 and incubated at 37 °C for 72 h. For shCtrl and shG9A cells, cells were seeded after dissociation without any treatment. Sphere-forming capacity of TICs was determined by the size of the spheres. After 72 h, images were taken and the cells were dissociated into single cells and counted for their cell proliferation capacity. For MTT assays, the treated and control cells were seeded in 96-well plates for 72 h. The cells were treated with 10 μl cell counting kit-8 (CCK-8) solution (Sigma, USA), incubated for 1–4 h and at the absorbance at 450 nm was recorded using microplate reader.

### Lentiviral production and infection

Stable G9A knocked down cells were established by creating shRNA expression cells lines for G9A. Lentiviral vectors expressing control shRNA (non-target) and G9A shRNA constructs (Dharmacon, USA) were transfected into 293T using Lipofectamine 3000 according to the manufacturer’s instructions. After 48 h of transfection, the supernatants containing lentivirus were harvested and used, to infect target cells with 6 μg/ml polybrene (Sigma, USA). After 72 h of transduction, cells were selected with 1.0 μg/ml puromycin for few days. The cells were harvested to determine the knockdown efficiency by western blot and qRT PCR.

### FOXP1 plasmid preparation and overexpression into TICs

FOXP1 ORF constructs were purchased from Dharmacon (USA) and were transiently overexpressed into LCSC4 and LCSC5 using lipofectamine 3000 using manufacturer’s instructions. For transfection, the TIC spheres were dissociated using StemPro accutase (ThermoFisher Scientific, USA) and 250 cells were transfected. After 72 h, 100,000 cells were seeded to carry out sphere formation and cell proliferation assays.

### Fluorescence activated cell sorting for CD133^+^ and CD133^−^ cells

Lung TICs spheres were dissociated into single-cell suspensions by StemPro Accutase (ThermoFisher Scientific, USA). The cells were stained with mouse anti-CD133-PE antibody (clone AC133; Miltenyi Biotec) and mouse. The cells were sorted by flowcytometry. The cells were centrifuged and the pellet was used to extract RNA and to prepare cell lysate for western blot analyses to examine G9A expression.

### Genomic DNA/RNA extraction

Genomic DNA was extracted from CICs pellets using PuneLink Genomic DNA kits (Life technologies, USA). Briefly, the cells were harvested from the growth medium by trypsinizing centrifugation homogenized using lysis buffer and incubated at 37 °C for 30 min followed by the addition of Proteinase K and RNase solution. The samples were then centrifuged and processed according to manufacturer’s instructions. The concentration of DNA and RNA was measured using *nanodrop2000* (Thermo Scientific, USA).

### Quantitative real-time reverse transcription-PCR

Total RNA extraction from TICs was carried out using RNeasy Kit (QIAGEN, USA). Briefly, the cell pellet was lysed using buffer RLT containing mercaptoethanol and mixed with 70% ethanol. The samples were then processed according to manufacturer’s instructions. The RNA concentration was measure by NanoDrop 2000 (ThermoFisher scientific, USA). cDNA was prepared using QuanTect reverse transcription kit (QIAGEN, USA). The qRT primers used for expression analyses are given in Supplementary Table 2B. B-actin gene was used as internal control for mRNA expression. Data were presented as the relative quantity of targets, normalized with respect to internal control, and relative to calibrator control sample.

### Cell lysate preparation, protein extraction Western blots, and immunohistochemistry

Western blot analyses were carried out using either Cell lysate or protein samples. To prepare cell lysate, the cell pellet was lysed using 2X SDS buffer (BD biosciences, USA). To extract protein from the tumors, the tumors were ground in dry ice, and mixed with 1X RIPA buffer (Invitrogen, USA).

Ten microliters of protein lysate or 20 μg of protein was separated by 4–12% sodium dodecyl sulphate polyacrylamide gel electrophoresis (PAGE) (Invitrogen, USA) and transferred onto polyvinylidene fluoride (PVDF) membrane (Invitrogen, USA), blocked with 5% non-fat milk in TBST, and blotted with the appropriate primary and secondary antibodies. Immunohistochemistry was carried out at molecular pathology laboratory of City of Hope National Medical Center. Quantification of Western blot images was carried out using ImageJ software [70]. The list of antibodies used and their details are given in Supplementary Table 3.

### Illumina BeadChip 450K HumanMethylation array

Two TICs (LCSC1 and LCSC4) with their corresponding G9A knocked down cells (control vs. G9A knocked down cells) were used to assess the genome-wide methylation profiling of > 850K individual CpG loci using Illumina Epitect BeadChip 850K HumanMethylation Array (850K-array). The 850K array profiling was carried out at Genomic Core facilities of City of Hope Medical Center. Chip processing was carried out based on 850K-array design according to manufacturer’s instructions. Signal intensities generated by Illumina *GenomeStudio* were converted to β values and *BeadStudio* software was used to remove biases between the Infinium I and II probes.

### Screening of candidate genes that have undergone DNA methylation changes following G9A knockdown

In order to generate an initial candidate list of genes that are either hypomethylated or hypermethylated in LCSC1 and 4 following G9A knockdown, we first looked for a global DNA methylation pattern in LCSC1 and 4 both in control (transfected with scrambled shRNA) and knocked down cells with shG9A#1 and shG9A#2. We initially screened individual CpG loci that were either hypermethylated or were hypomethylated in knocked down TICs compared to scrambled control TICs using unsupervised clustering with the help of cluster 3.0 software. The differentially methylated probes based on unsupervised clustering were further refined for which individual CpGs with β value of ≥ 0.35 was considered as methylated and CpGs with β value of ≤ 0.35 was considered as unmethylated. We then curated individual hypomethylated loci that had average β values < 0.35 in knocked down cells with shG9A#1 and shRNA#2 and β values of ≥ 0.35 in cells transfected with scrambled shRNA. In addition, each differentially methylated CpGs were had a β value differences of ≥ 0.15 between control cells and knocked down cells. The genes corresponding to differentially methylated CpGs were taken further as differentially methylated candidate genes for downstream validation.

### RNA seq analyses

RNA sequencing libraries were prepared with Kapa RNA mRNA HyperPrep kit (Kapa Biosystems, USA) according to the manufacturer’s protocol. The sequencing libraries were validated with the Agilent Bioanalyzer DNA High Sensitivity Kit and quantified with Qubit. The libraries were sequenced on Illumina HiSeq 2500 with SR V4 Kit with the single read mode of 51cycle of read1 and 7 cycles of index read. Real-time analysis (RTA) 2.2.38 software was used to process the image analysis and base calling.

RNA sequencing data analyses were performed using the HISeq 2500 (Illumina, USA) according to manufacturer’s instructions. Briefly, 500 ng of RNA was converted into cDNA library, which was then sequenced using Illumina Hiseq2500 with single read 40 bp based on manufacturer’s instructions. Raw sequence reads were mapped to the human genome (hg19) using STAR_2.5.3a [71] and the frequency of genes was counted using HTSeq-0.6.1p1 [72]. The raw counts were then normalized using the trimmed mean of M values (TMM) method and compared using Bioconductor package “edgeR 3.16.5” [73]. Reads per kilobase per million (RPKM) mapped were also calculated from the raw counts. Differentially expressed genes were identified if RPKM ≥ 1 in at least one sample, fold change ≥ 2, and *P* ≤ 0.05.

### Bisulfite conversion of DNA

Bisulfite conversion of genomic DNA from TICs was carried out using the *Epitect Bisulfite kit* (QIAGEN, USA). Briefly, 500 ng DNA from TICs was added to 200 μl sterile PCR tube. The samples were mixed up with 85 μl bisulphite conversion reagent (bisulphite mix), 85 μl DNA protect buffer, and RNase-free water making up total volume of 140 μl. The samples were then processed according to manufacturer’s instructions. For positive controls, fully methylated, positive controls were generated by incubating genomic DNA with DNA methyltransferases in the presence of S-Adenosyl methionine (SAM) (New England bio lab, USA) for 2 h at 37 °C prior to bisulfite conversion.

### Experimental validation of methylation status of individual genes

The methylation status of each gene corresponding to differentially methylated probes was determined by bisulphite sequencing. First, the genes were PCR-amplified using combined bisulphite and restriction analyses (CoBRA). CoBRA primers were designed based on the standard primary designing criteria used in analyzing bisulphite converted DNA [74–76]. The CoBRA primers used for bisulphite sequencing are given in Supplementary Table 2A.

### Tumorigenicity assay

Tumorigenic potential of cells in vivo following G9A knockdown was assessed by transplanting TICs into the NOD-SCID mice. The TIC spheres (both with scrambled shRNA and shG9A) were dissociated with stem cell accutase and the dissociated cells (10^3^ to 10^4^ cells) population from spheres were suspended in Matrigel (BD Biosciences, USA) and injected subcutaneously into the right flank of four NOD-SCID mice respectively. After 4 weeks of the injection, the tumor size was measured every week. Mice were euthanized 60 days after the injection. In addition, the dissociated single cells (100,000 cells) from wild-type TIC spheres (LCSC2 and LCSC4) were injected subcutaneously into the NOD-SCID mice. After 8 weeks transplantation, mice were divided into two groups. Each group was treated either by vehicle or by G9A inhibitor UNC0642 (10 mg/kg) for 8 weeks. After the treatment mice were euthanized and the tumor weight was measured. The tumors were fixed using 10% formaldehyde solution for downstream investigation. All mice in this study were in a pathogen-free environment. The use of animals was approved by the IRB-17196 of City of Hope National Medical Center and all applicable institutional and governmental regulations concerning the ethical use of animals were followed.

### Chromatin immunoprecipitation assay

LCSC4 and LCSC5 cells grown on culture were cross-linked to chromatin by adding 1% formaldehyde to each of the cell suspension. The samples were incubated at room temperature for 10 min, and cross linking was stopped by adding of 125 mM glycine and incubating at room temperature for 5 min. Cell were pelleted and washed with cold PBS, resuspended in cell lysis buffer containing 1× protease inhibitor, and incubated on ice for 15 min. Cell suspension was centrifuged and resuspended in 0.5 ml nuclear lysis buffer and sonicated to generate chromatin fragments. Samples were centrifuged, and 450 μl dilution buffer was added to each 50 μl supernatant making up 500 μl chromatin aliquots. Then, 20 μl protein A agarose was added to each sample, and followed by IGG (for negative control) and H3K9Me2 antibody which were incubated at 4 °C overnight with rotation. Protein A agarose slurry containing immune complexes were collected, and washed with rotation with cold low-salt buffer, high-salt buffer, LiCl buffer, and TE buffer each for 5 min. Bound complexes were eluted off the beads in 100 μl ChiP elution buffer with proteinase K solution. The samples were incubated at 65 °C for 4 h and 95 °C for 10 min to reverse formaldehyde crosslinking. The beads were separated using magnetic separator and DNA contained in supernatant was column purified using following manufacturer’s instructions. The QPCR was carried out using primers designed at the promoter region of the respective target genes. QPCR primers used are given in Supplementary Table 2C.

### Survival analyses

Survival analyses of lung cancer patients were carried out to investigate the correlation of expression of candidate genes with clinical prognosis using online KM-plotter tools [40]. KM-plotter, an online tool, measures the prognostic indication of 22,277 genes based on gene expression data and survival information of 1735 non-small cell lung cancer patients from Affymetrix HGU133A and HGU133 microarrays datasets of ten independent datasets from Gene Expression Omnibus (GEO), CaBIG, and The Cancer Genome Atlas (TCGA) [77]. The tool measures the prognostic significance by segregating patients on high or low expressing groups based on median/lower/upper quartile as a cut off to calculate a statistical significance of gene expression with respect to patient survival. Logrank *P* < 0.05 was considered statistically significant.

### Statistical analysis

Fisher’s exact test was used to determine the statistical significance of methylation between the TICs transfected with scrambled shRNA and shG9A#1 and shG9A#2. *P* < 0.05 was considered statistically significant. Similarly, statistical test was carried out on RNA sequencing for each gene between control and G9A knocked down samples using R package.

## Supplementary information

**Additional file 1:.** Supplementary Figure 1. (A) Patient-derived TICs isolated and cultured used in the experiments. (B) FACS-sorted CD133-positive cells (LCSC4) have higher sphere forming capacity compared to CD133 negative cells. (C) Quantification of western blots for CD133 level in CD133+ and CD133- cells as well as in G9A knocked down cells and (D) H3K9me2 level in G9a knocked down cells. E) Cell proliferation capacity of TICs was measured by taking OD 450nm after the cells were treated with UNC0642 for 72 hours. (For t-test: *= P<0.05, **=P<.01 And ***=P<0.001).

**Additional file 2: **Supplementary Figure 2. G9A suppression using shRNAi contributes to Genome-wide methylome and transcriptome changes in patient derived TICs from NSCLC. An initial unsupervised clustering of Genome-wide methylation profiling (850K methylation array) data shows that G9A contributes to Genome wide methylation changes in TICs *i.e.* LCSC1 (A), and LCSC4 (B) following G9A knockdown. (C) Individual methylation profiling of candidate genes that were hypomethylated and upregulated commonly in LCSC1 and 4.

**Additional file 3:.** Supplementary Figure 3. FOXP1 prompter region amplified and the primers used in order validate methylation status for bisulphite sequencing. (A) CpG island promoter region of FOXP1 and primers designed to CoBRA amplify this region to validate methylation status using bisulphite sequencing. (B) Methylation status of SP5 as an additional representative example.

**Additional file 4:.** Supplementary Figure 4. Quantification of western blot assays for G9A and its target genes following G9A knockdown and treatment of TICs by G9A inhibitor using Image J. (For t-test: *= P<0.05, **=P<.01 And ***=P<0.001).

**Additional file 5: **Supplementary Figure 5. High-expression of candidate genes (A) *DPP4*, *(B) STAMBPL1*, *and* (C) *ROBO1* correlates to better clinical outcomes of patients in lung cancers (n=number of patients whose mRNA for respective genes were used for Kaplan Meier analyses).

**Additional file 6: **Supplementary Figure 6. Expression status (qRT PCR) of G9A target genes to examine their mRNA level after the cells were treated by UNC0642, 5-aza-2’-DC and combined with UNC0642 and 5-aza-2’-DC. Expression level of (A) DPP4, (B) SP5 and (C) *CDYL2* in *LCSC4* and *LCSC5*. (D, E) QPCR following chromatin immunoprecipitation assays on G9a target genes *CDYL2*, and *DPP4*. (For t-test: *= P<0.05, **=p<.01 and ***=p<0.001).

**Additional file 7:.** Supplementary Figure 7. Quantification of western blot assays for A) FOXP1, B) CD133, C) CD44, D) ALDH1A3 and E) H3K9Me2 following FOXP1 overexpression in LCSC2, LCSC4 and LCSC5. Quantification was carried out using Image J. (For t-test: *= P<0.05, **=P<.01 And ***=P<0.001).

**Additional file 8:.** Sup Tables.

## Abbreviations

CoBRA: Combined bisulphite and restriction analyses
TCGA: The Cancer Genome Atlas
CSCs: Cancer stem cells
DAVID: The databases for annotation, visualization and integrated discovery
DNMT1: DNA methyltransferaase-1
EHMT2: Eukaryotic histone methyltransferases 2
IHC: Immunohistochemistry
KM: Kaplan-Meier
MI: Methylation Index
NSCLC: Non-small cell lung cancer
RPKM: Reads per kilobase per million
TIC: Tumor-initiating cells
