# Supplementary figures and images for: G9a regulates tumorigenicity and stemness through genome-wide DNA methylation reprogramming in non-small cell lung cancer
Source: Clin Epigenetics. 2020 Jun 17;12:88. doi: 10.1186/s13148-020-00879-5 (PMC7302379; doi:10.1186/s13148-020-00879-5)

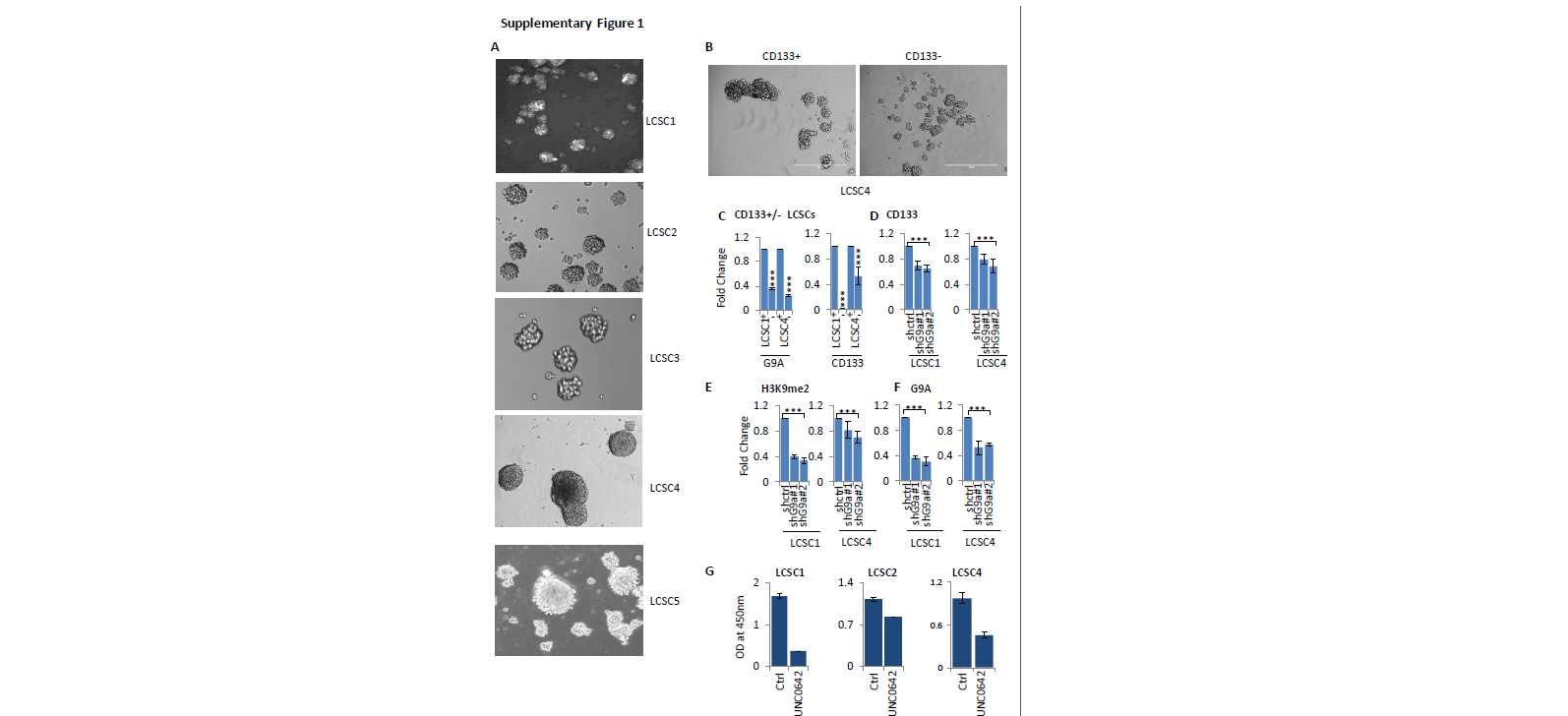

Supplement: Supplementary file 1 — Additional file 1:. Supplementary Figure 1. (A) Patient-derived TICs isolated and cultured used in the experiments. (B) FACS-sorted CD133-positive cells (LCSC4) have higher sphere forming capacity compared to CD133 negative cells. (C) Quantification of western blots for CD133 level in CD133+ and CD133- cells as well as in G9A knocked down cells and (D) H3K9me2 level in G9a knocked down cells. E) Cell proliferation capacity of TICs was measured by taking OD 450nm after the cells were treated with UNC0642 for 72 hours. (For t-test: *= P<0.05, **=P<.01 And ***=P<0.001). [file 13148_2020_879_MOESM1_ESM.png]

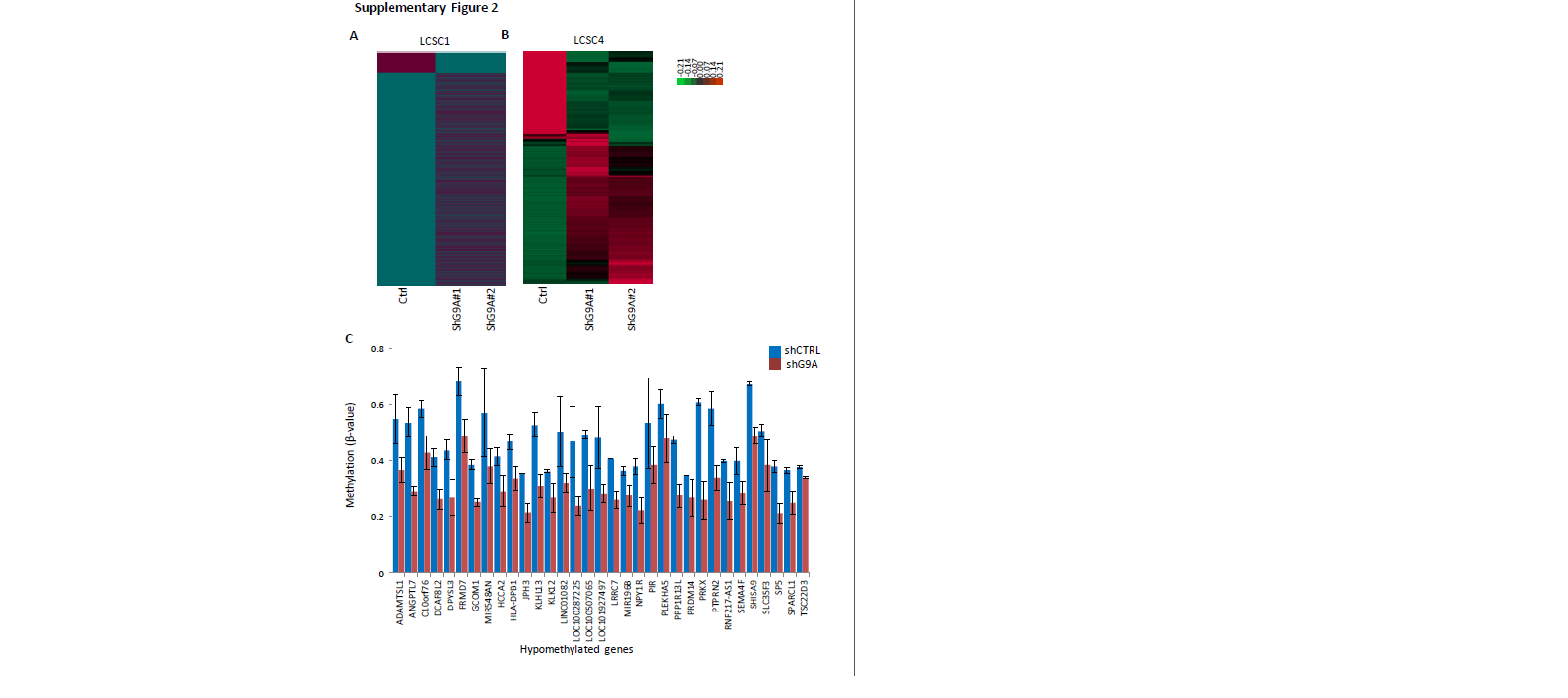

Supplement: Supplementary file 2 — Additional file 2: Supplementary Figure 2. G9A suppression using shRNAi contributes to Genome-wide methylome and transcriptome changes in patient derived TICs from NSCLC. An initial unsupervised clustering of Genome-wide methylation profiling (850K methylation array) data shows that G9A contributes to Genome wide methylation changes in TICs i.e. LCSC1 (A), and LCSC4 (B) following G9A knockdown. (C) Individual methylation profiling of candidate genes that were hypomethylated and upregulated commonly in LCSC1 and 4. [file 13148_2020_879_MOESM2_ESM.png]

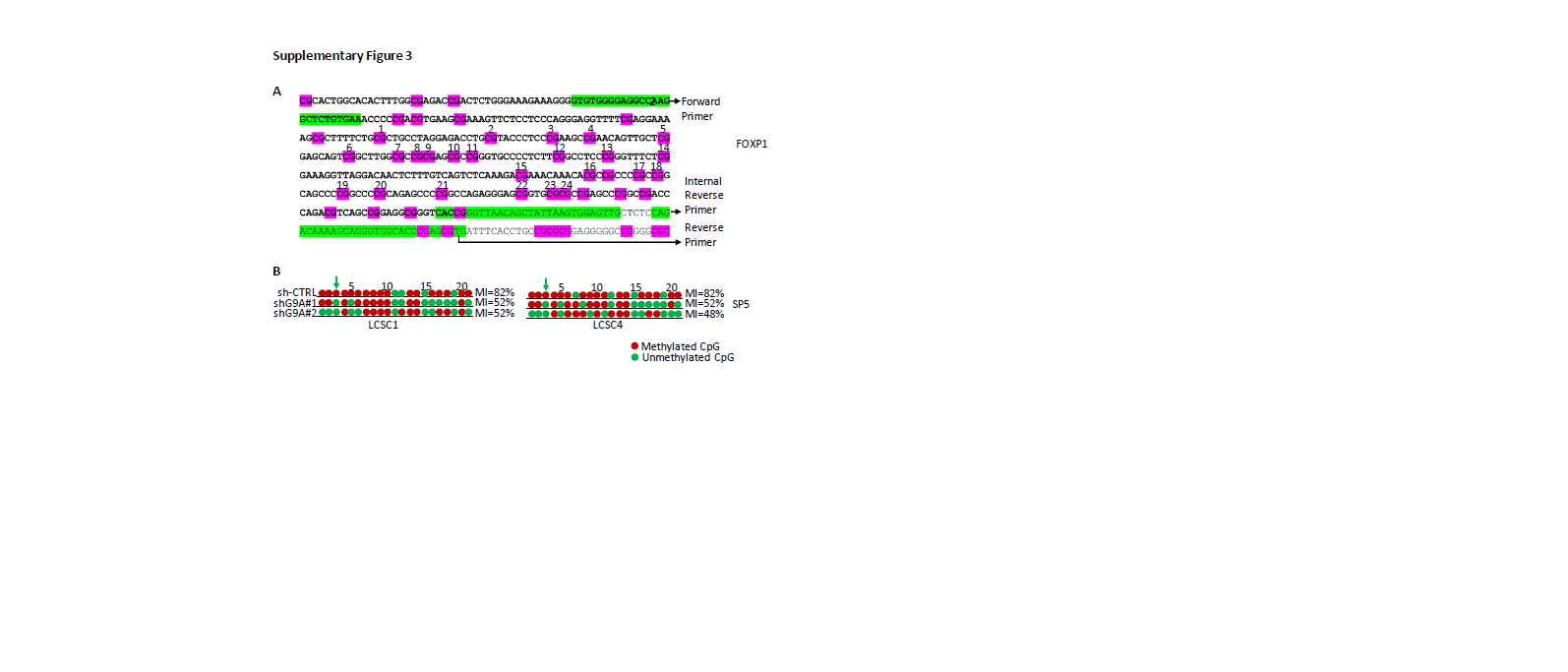

Supplement: Supplementary file 3 — Additional file 3:. Supplementary Figure 3. FOXP1 prompter region amplified and the primers used in order validate methylation status for bisulphite sequencing. (A) CpG island promoter region of FOXP1 and primers designed to CoBRA amplify this region to validate methylation status using bisulphite sequencing. (B) Methylation status of SP5 as an additional representative example. [file 13148_2020_879_MOESM3_ESM.png]

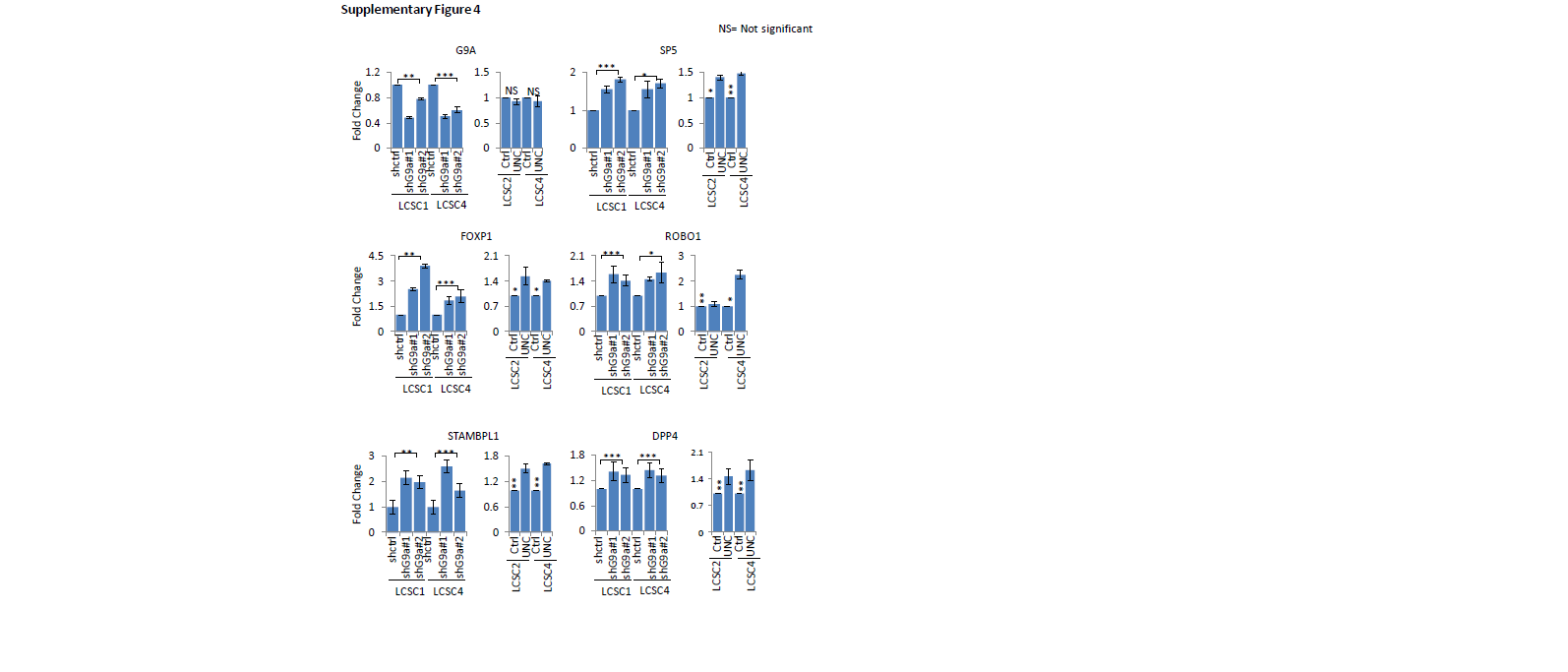

Supplement: Supplementary file 4 — Additional file 4:. Supplementary Figure 4. Quantification of western blot assays for G9A and its target genes following G9A knockdown and treatment of TICs by G9A inhibitor using Image J. (For t-test: *= P<0.05, **=P<.01 And ***=P<0.001). [file 13148_2020_879_MOESM4_ESM.png]

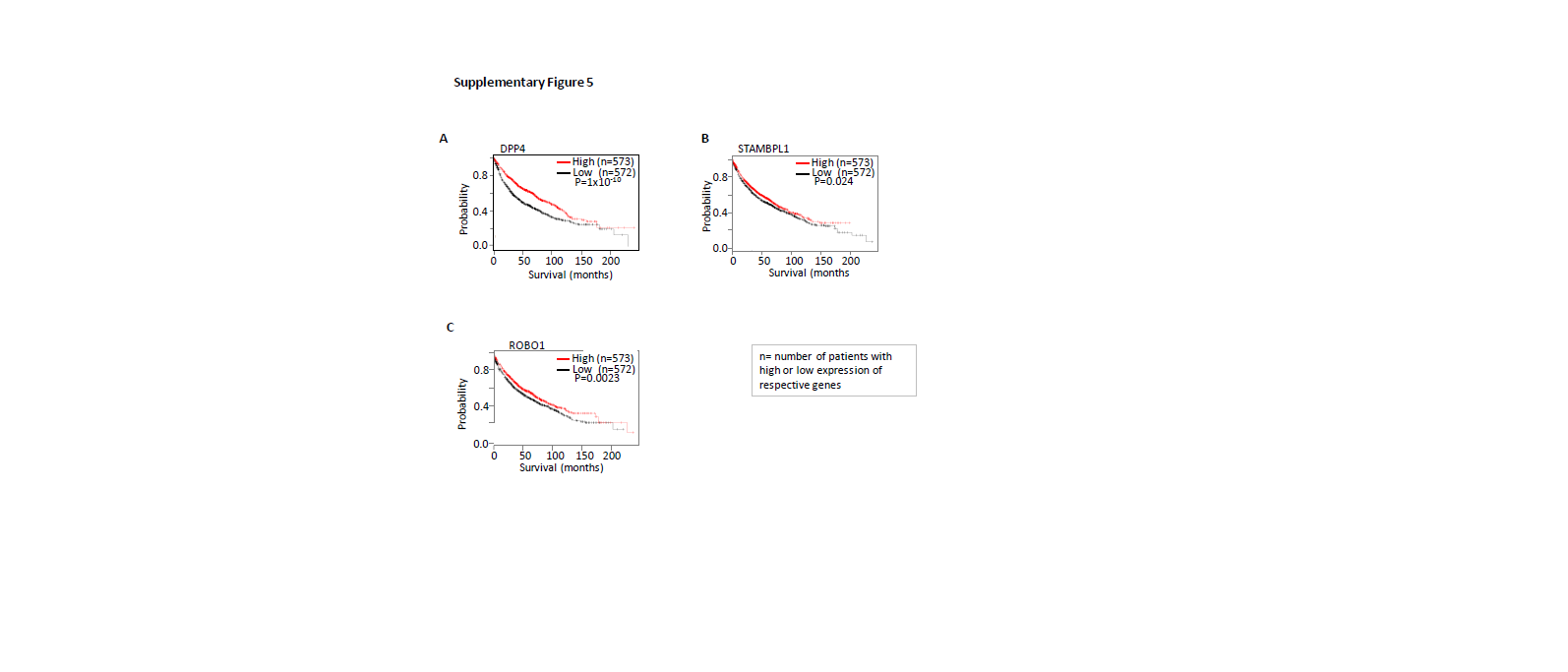

Supplement: Supplementary file 5 — Additional file 5: Supplementary Figure 5. High-expression of candidate genes (A) DPP4, (B) STAMBPL1, and (C) ROBO1 correlates to better clinical outcomes of patients in lung cancers (n=number of patients whose mRNA for respective genes were used for Kaplan Meier analyses). [file 13148_2020_879_MOESM5_ESM.png]

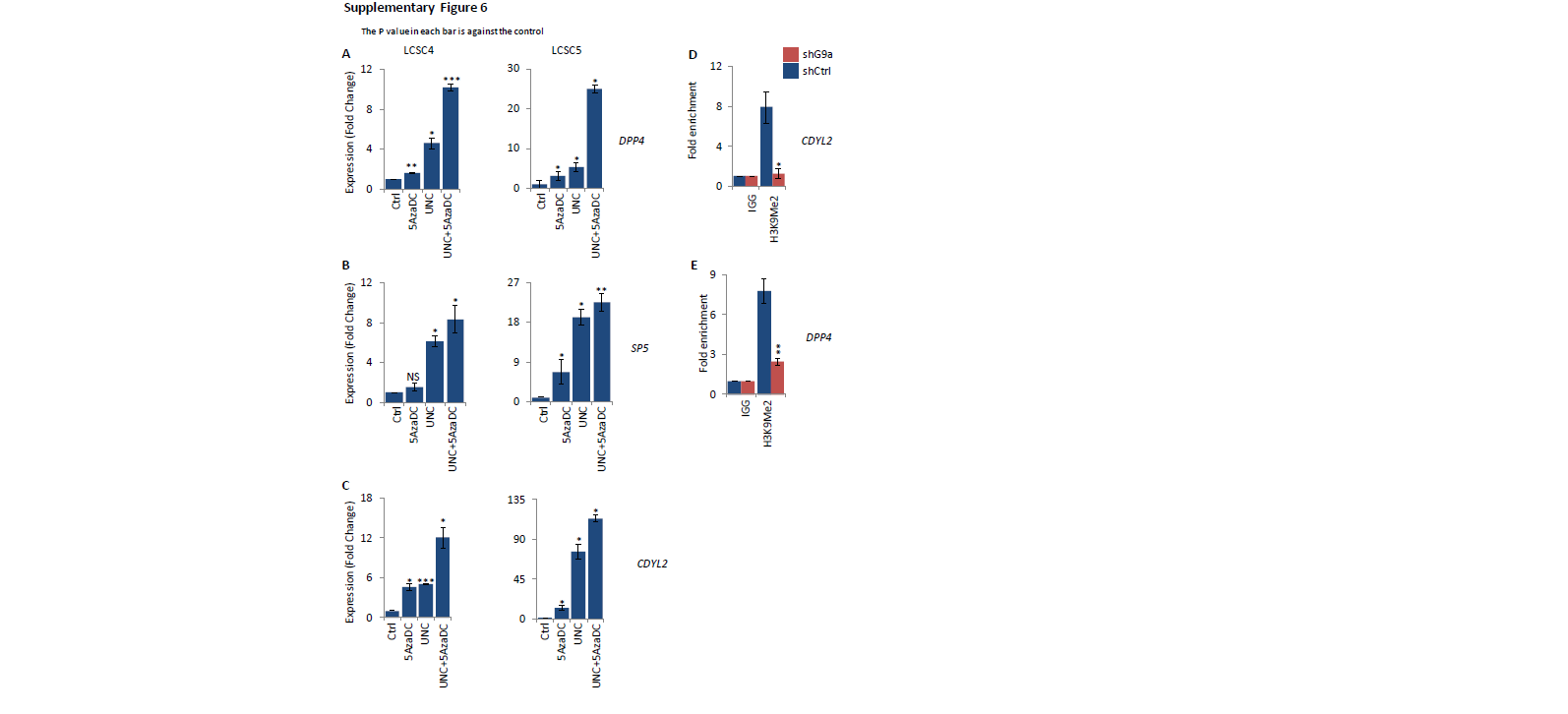

Supplement: Supplementary file 6 — Additional file 6: Supplementary Figure 6. Expression status (qRT PCR) of G9A target genes to examine their mRNA level after the cells were treated by UNC0642, 5-aza-2’-DC and combined with UNC0642 and 5-aza-2’-DC. Expression level of (A) DPP4, (B) SP5 and (C) CDYL2 in LCSC4 and LCSC5. (D, E) QPCR following chromatin immunoprecipitation assays on G9a target genes CDYL2, and DPP4. (For t-test: *= P<0.05, **=p<.01 and ***=p<0.001). [file 13148_2020_879_MOESM6_ESM.png]

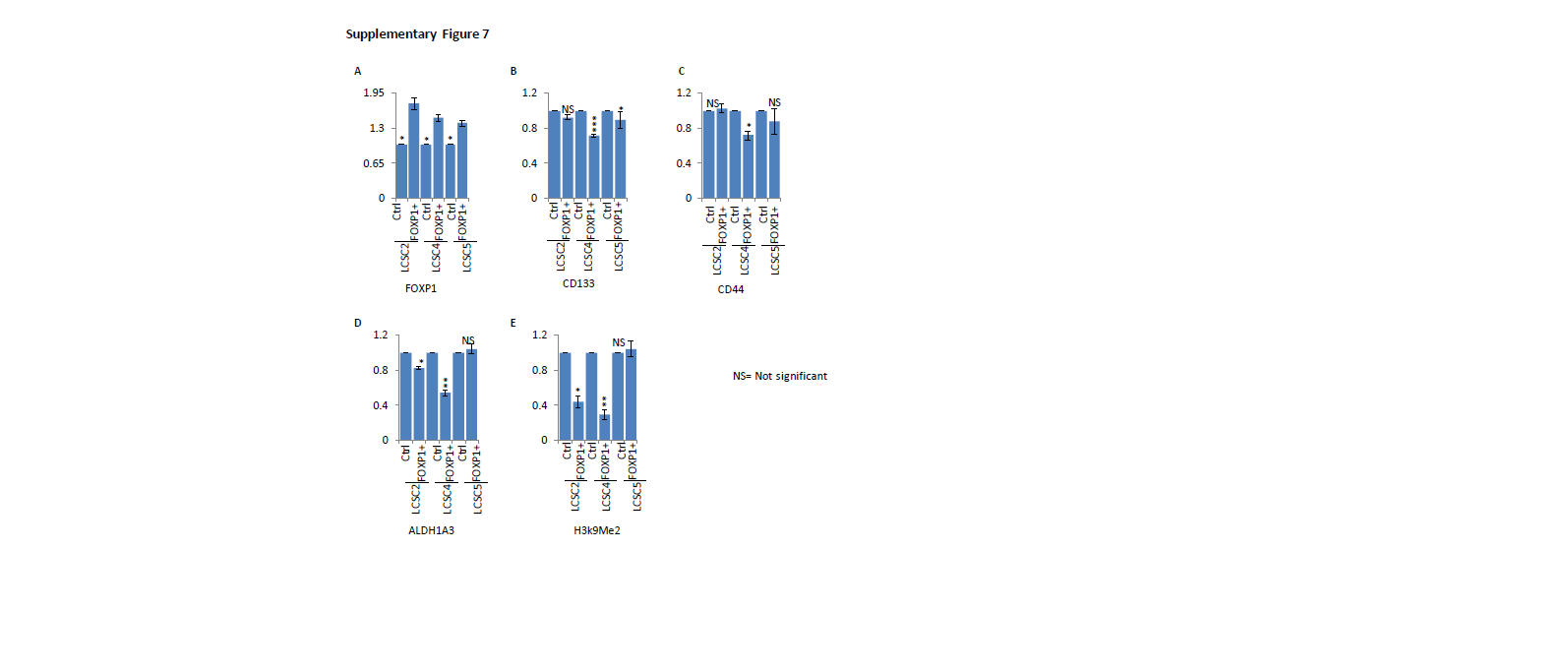

Supplement: Supplementary file 7 — Additional file 7:. Supplementary Figure 7. Quantification of western blot assays for A) FOXP1, B) CD133, C) CD44, D) ALDH1A3 and E) H3K9Me2 following FOXP1 overexpression in LCSC2, LCSC4 and LCSC5. Quantification was carried out using Image J. (For t-test: *= P<0.05, **=P<.01 And ***=P<0.001). [file 13148_2020_879_MOESM7_ESM.png]
